# Rituximab for the treatment of membranous nephropathy in a patient with ankylosing spondylitis: a case report

**DOI:** 10.3389/fmed.2026.1757906

**Published:** 2026-03-13

**Authors:** Rongzhen Zhong, Li Su, Hongxia Li, Hongyan Zhu, Zhiqing Xiao

**Affiliations:** 1 Department of Nephrology, The Seventh Medical Center, Chinese PLA General Hospital, Beijing, China; 2 Department of Pathology, The Seventh Medical Center, Chinese PLA General Hospital, Beijing, China

**Keywords:** ankylosing spondylitis (AS), biological agents (BAs), membranous nephropathy (MN), membranous nephropathy in a patient with ankylosing spondylitis, nephrotic syndrome (NS), rituximab

## Abstract

Ankylosing spondylitis (AS) is a chronic inflammatory disease primarily affecting the axial skeleton. Renal involvement is uncommon in AS, occurring in only 5–13% of patients, with membranous nephropathy (MN) being particularly rare, with only 10 cases reported in PubMed to date. We report the case of a 37-year-old man with an 11-year history of AS who developed nephrotic syndrome during the stable phase of his disease. Renal biopsy confirmed MN, revealing IgG and C3 deposition along the glomerular capillary walls and subepithelial electron-dense deposits. After excluding secondary causes, rituximab was selected as the initial immunosuppressive therapy. Six months after treatment, the patient’s 24-h urinary protein excretion decreased from 12.9 g to 836 mg, and serum albumin increased from 21.4 g/L to 35.4 g/L, achieving partial remission. This case provides additional clinical evidence of a potential association between AS and MN and demonstrates the efficacy and safety of rituximab in this rare clinical scenario, offering insight into potential treatment strategies.

## Introduction

Ankylosing spondylitis (AS) is a chronic autoimmune disease primarily involving the sacroiliac joints and spine and may be accompanied by extra-articular manifestations (1, 2). The kidneys are among the commonly affected organs, with IgA nephropathy being the most frequent renal pathology.

Membranous nephropathy (MN) is a common cause of nephrotic syndrome in adults and is characterized by the deposition of immune complexes in the subepithelial space of the glomerular basement membrane. It can be classified as primary or secondary, with secondary MN often associated with infections, autoimmune diseases, drugs, or malignancies.

The co-occurrence of AS and MN is extremely rare in clinical practice. A recent retrospective study of renal biopsies in 62 patients with AS reported IgA nephropathy in 74.2% of cases, whereas MN accounted for only 3.2% (3). To date, only 10 such cases have been reported in the PubMed database (Table 1) (4, 5).

**Table 1:** Case reports of the association between membranous nephropathy and ankylosing spondylitis.

Authors	Age/sex	Associated rheumatoid arthritis or tumors	BASDAI score	Drugs administered	Treatment plan	Follow-up
Lemmer and Irby (21)	51/M	+	NA	NSAIDs, gold salts	NA	NA
Botey et al. (22)	42/M	–	NA	Steroids	NA	NA
Serrano Comino et al. (23)	35/M	+	NA	NSAIDs, gold salts	Discontinuation of NSAIDs	RFT normal at 3 years
Efstratiadis et al. (24)	50/M	–	NA	NSAIDs	NA	Died, 40 days (MI)
Gupta et al. (14)	29/M	–	NA	NSAIDs (intermittent)	Prednisone and cyclophosphamide	Partial remission at 1.5 years
Kaushik et al. (8)	60/F	–	NA	NSAIDs, methotrexate, etanercept	Discontinuation of etanercept	RFT normal at 1 year
Chen et al. (25)	44/F	–	NA	NSAIDs	Discontinuation of NSAIDs and initiation adalimumab (TNF-*α*)	Partial remission for 3 months
Lin et al. (26)	64/F	Thymoma	6.08	NSAIDs	Methylprednisolone (500 mg) for 3 consecutive days, followed by oral methylprednisolone 40 mg QD	Partial remission at 6 months
Tohma et al. (4)	41/F	–	2.40	NSAIDs, sulfasalazine	Discontinuation of NSAIDs and initiation of ACE inhibitor, prednisolone, and cyclosporine	Partial remission at 2 months
Zhao et al. (5)	47/M	–	NA	NSAIDs (intermittent)	Prednisolone and cyclophosphamide	RFT normal at 2 years
Present case	37/M	–	2.60	NSAIDs, sulfasalazine, etanercept (all intermittent)	ARB, rituximab	RFT normal at 1 year

NSAIDs, non-steroidal anti-inflammatory drugs; NA, not available; MI, myocardial infarction; RFT, renal function tests; ACE, angiotensin-converting enzyme; ARB, angiotensin II receptor blocker; TNF-α, Tumor Necrosis Factor-Alpha.

Notably, most reported cases had confounding factors, such as long-term use of non-steroidal anti-inflammatory drugs (NSAIDs) or biologic agents, making it difficult to establish a causal relationship between AS and MN. Whether MN represents a rare renal manifestation of AS or a mere coincidental coexistence remains unclear.

This report describes a 37-year-old male patient with AS who developed nephrotic syndrome during a stable phase of his disease, with renal biopsy confirming MN. Common secondary causes were excluded, and atypical pathological features were observed, providing additional information for exploring the potential association between AS and MN. Importantly, this is the first reported case in which rituximab was used as the initial immunosuppressive therapy with a favorable outcome, offering new perspectives and insight into the management of such rare patients’ clinical scenarios.

## Case report

A 37-year-old man was admitted to our department due to “symmetrical edema of both ankles after exertion for 2 months.” Two months prior, the patient developed symmetrical pitting edema of both ankles following physical exertion, which progressively worsened and extended to both lower limbs. Laboratory tests at a local hospital revealed albumin of 29 g/L and urine protein 4+, prompting referral to our unit for further management.

**Figure 1:**
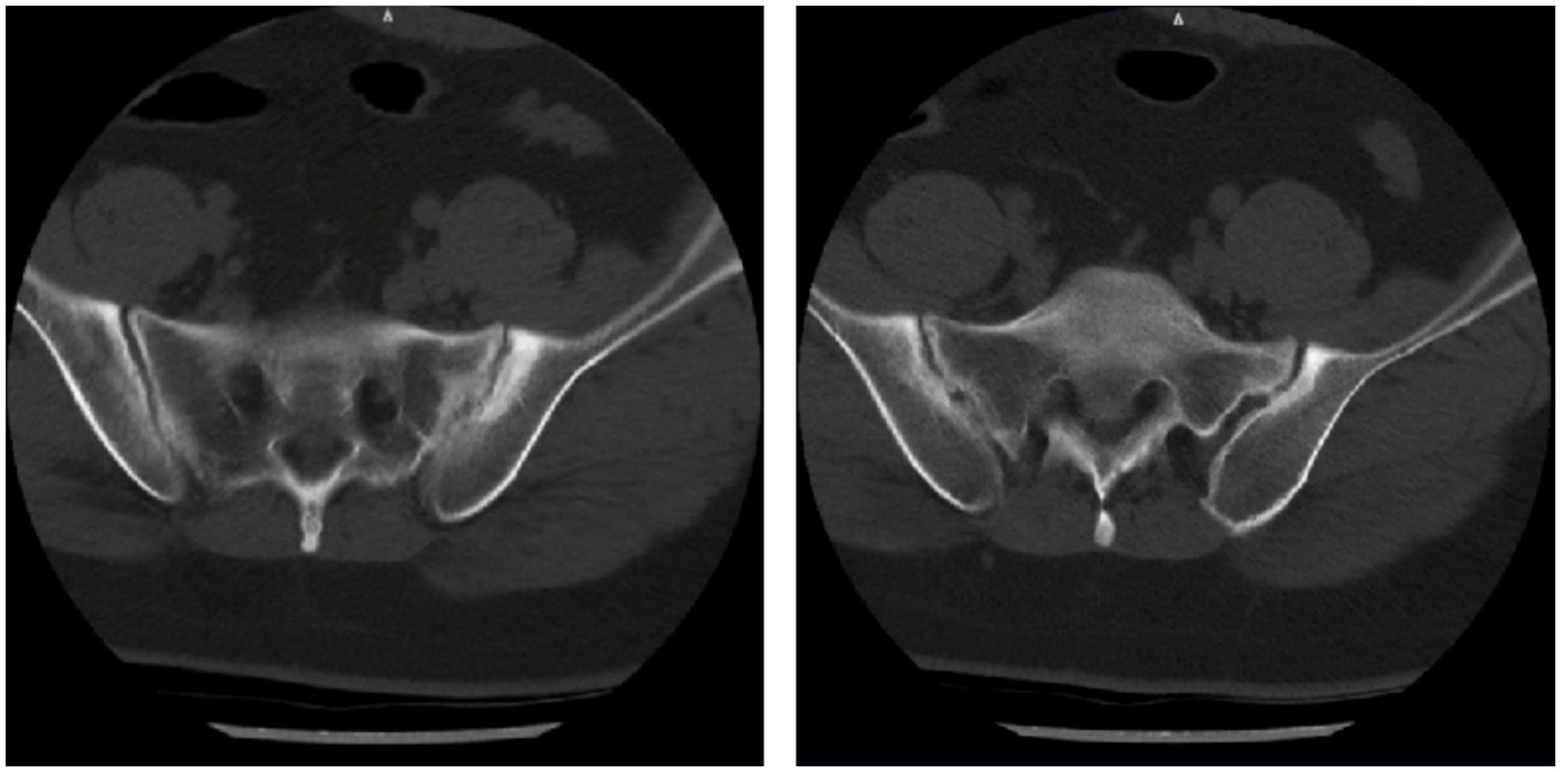
Lumbosacral spiral CT imaging: bilateral sacroiliac joints show blurred articular surfaces, serrated edges, and narrowed joint spaces, consistent with ankylosing spondylitis.

The patient had been diagnosed with AS 11 years earlier based on bilateral knee and ankle joint pain and swelling, and a positive HLA-B27 test. Initial imaging records were unavailable due to the elapsed time; a recent CT scan showed bilateral sacroiliitis (Figure 1). Following the AS diagnosis, he received etanercept (50 mg/week, subcutaneously) for 3 months, along with oral sulfasalazine and ibuprofen for 2 years; all medications had been discontinued 9 years before the current presentation.

Half a month after the onset of the current symptoms, suspecting an AS flare, the patient revisited a local hospital, where tests showed albumin 30.1 g/L and urine protein 4+. He was restarted on sulfasalazine (1 g/day) and etanercept (50 mg/week) for 1 month but showed no improvement and subsequently discontinued these medications.

On admission, physical examination revealed a height of 175 cm, a weight of 96 kg, a BMI of 31.3 kg/m^2^, and pitting edema of both lower limbs, with no other positive findings. Computed tomography (CT) scans of the chest and abdomen revealed no evidence of malignancy. The Bath Ankylosing Spondylitis Disease Activity Index (BASDAI) score was 2.6. He had no history of hypertension, diabetes, or hepatitis. Family history indicated that his uncle and younger brother had AS, but there was no family history of kidney disease. He had a 10-year smoking history (10 cigarettes/day), no alcohol consumption, and no history of exposure to toxins or radiation.

Laboratory investigations showed a serum creatinine of 66.0 μmol/L and an estimated glomerular filtration rate (eGFR) of 117.4 mL/min/1.73 m^2^. Serum albumin was 21.4 g/L and 24-h urinary protein excretion was 12.9 g. Low-density lipoprotein (LDL) cholesterol was 452.0 mg/dL. Bone metabolism assessment revealed osteoporosis, with a 25-hydroxyvitamin D level of 3 ng/mL. Multiple serum tests for anti-phospholipase A2 receptor (anti-PLA2R) antibodies were negative.

Urinalysis showed specific gravity 1.032, urinary protein 5 g/L, and urinary red blood cells 1.1/HPF. Erythrocyte sedimentation rate was 76 mm/h. The serum quantitative IgG1 level was reduced to 2990.0 mg/L (adult reference range: 3824.0–9286.0 mg/L). Levels of total IgG, IgG3, IgG4, IgA, and complement components C3 and C4 were within normal limits. Tests for hemoglobin A1c, thyroid function, C-reactive protein, procalcitonin, respiratory pathogens, complete blood count, T-cell detection for *Mycobacterium tuberculosis* infection, hepatitis B virus, hepatitis C virus, syphilis, HIV, tumor markers, antinuclear antibodies (ANAs), anti-double-stranded DNA (anti-dsDNA), anti-glomerular basement membrane (anti-GBM) antibodies, and anti-neutrophil cytoplasmic antibodies (ANCAs) were all negative.

**Figure 2:**
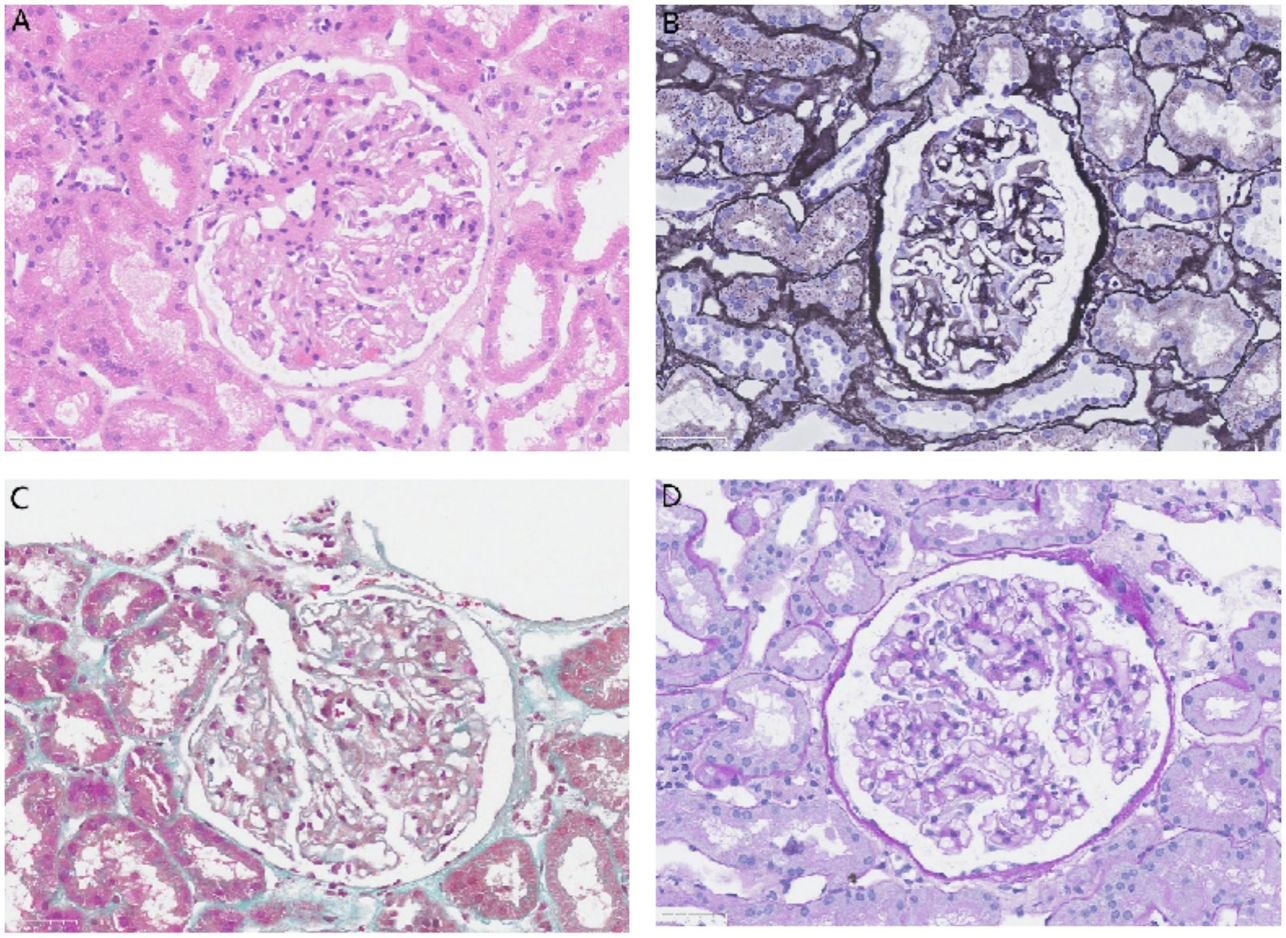
Light microscopy: glomeruli show diffuse vacuolar degeneration of the capillary basement membrane, segmental mild thickening, mild diffuse mesangial cell and matrix hyperplasia, and subepithelial eosinophilic deposits. Renal tubules exhibit epithelial granular and vacuolar degeneration, focal atrophy, focal lymphocytic and mononuclear cell infiltration in the interstitium, and mild thickening of small arteries. **(A)** Hematoxylin and eosin stain (×400), **(B)** Periodic acid–silver methenamine stain (×400), **(C)** Masson’s trichrome stain (×400), and **(D)** Periodic acid–Schiff stain (×400).

To determine the etiology of the nephrotic syndrome, an ultrasound-guided renal biopsy was performed. Light microscopy examination of 12 glomeruli revealed diffuse vacuolar degeneration of the glomerular basement membrane, segmental mild thickening, mild diffuse mesangial cell and matrix hyperplasia, and subepithelial eosinophilic deposits. Renal tubules showed epithelial granular and vacuolar degeneration, focal atrophy, focal lymphocytic and mononuclear cell infiltration in the interstitium, and mild thickening of small arteries (Figure 2).

**Figure 3:**
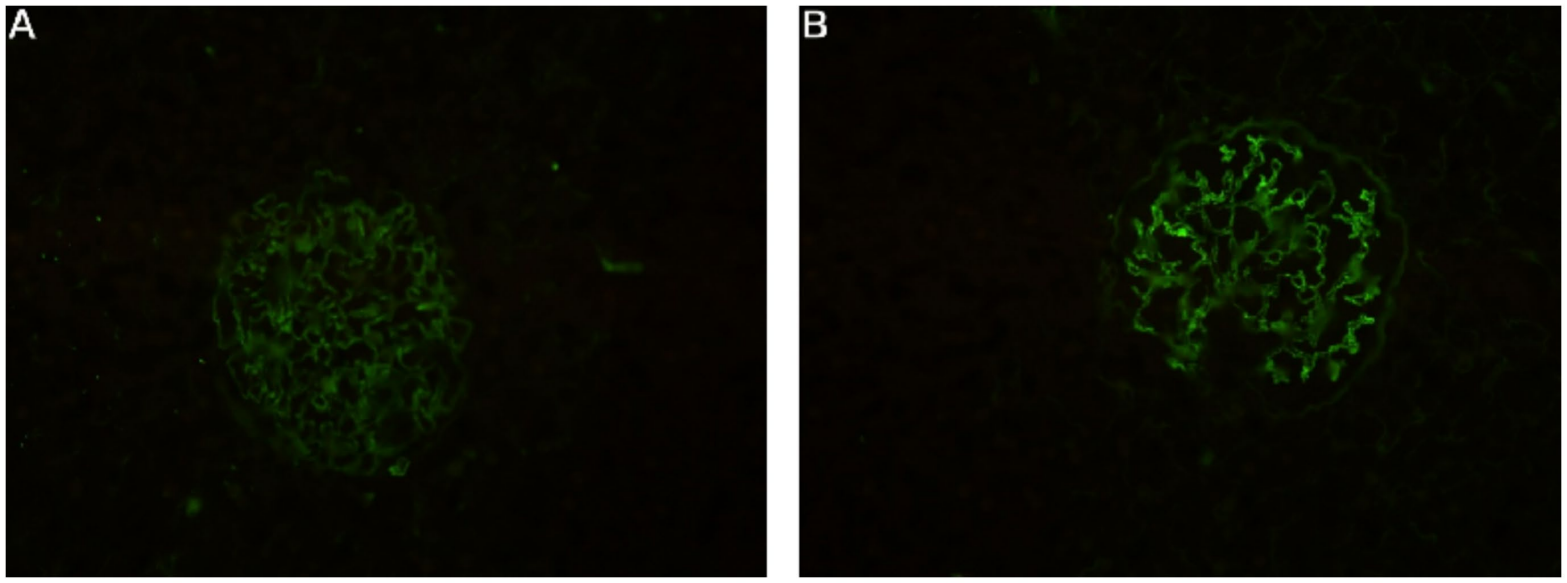
Immunofluorescence: strong (+++) granular capillary wall staining for **(A)** C3 and **(B)** IgG.

**Figure 4:**
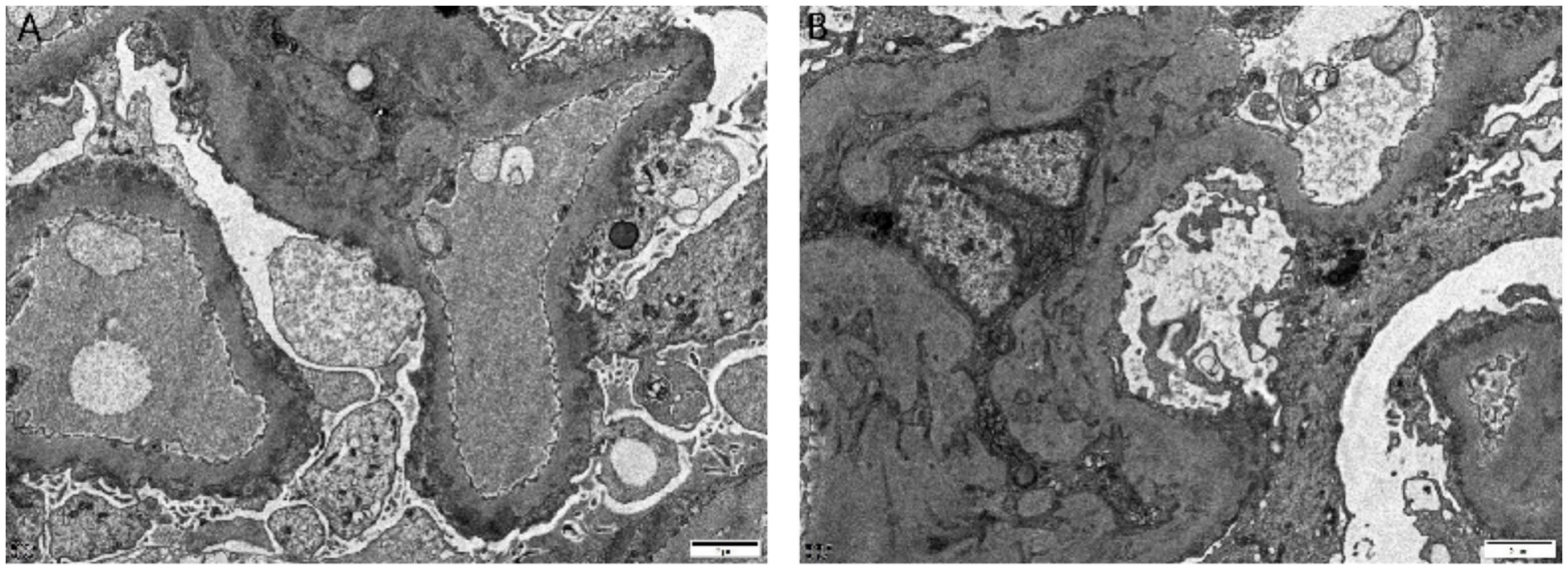
Electron microscopy: segmental thickening of the glomerular basement membrane with spike formation, subepithelial, intramembranous, and segmental mesangial electron-dense deposits, and extensive effacement of podocyte foot processes. Tubular epithelial cells show vacuolar degeneration and increased lysosomes **(A)** (×5,000) and **(B)** (×20,000).

Immunofluorescence demonstrated strong (+++) granular capillary wall staining for IgG and C3, while IgA, IgM, and C1q were negative (Figure 3). Electron microscopy revealed segmental thickening of the glomerular basement membrane with spike formation, subepithelial, intramembranous, and mesangial electron-dense deposits, and extensive effacement of podocyte foot processes. Tubular epithelial cells showed vacuolar degeneration and increased lysosomes (Figure 4). Additional immunohistochemical staining of the renal tissue showed positive staining for IgG along the capillary walls and in the mesangial areas, whereas IgG4 staining was negative.

Supportive management included diuretics for edema reduction, irbesartan 150 mg/day for proteinuria reduction, atorvastatin 20 mg/day for lipid control, rivaroxaban 10 mg/day for anticoagulation and thrombosis prophylaxis, and calcitriol 0.25 μg/day for osteoporosis prevention. As the patient declined corticosteroid therapy and considering the increased risk of adverse effects due to obesity, rituximab was selected after detailed discussion (1 g administered on days 1 and 15).

At the 3-month follow-up, lower extremity edema had resolved. Laboratory tests revealed serum creatinine of 62.5 μmol/L, eGFR of 120.1 mL/min/1.73 m^2^, 24-h urinary protein excretion of 1853 mg, serum albumin of 32.2 g/L, and an absolute B-cell count of 1/μL. By the 6-month follow-up, 24-h urinary protein had further decreased to 836 mg, with a corresponding increase in serum albumin to 35.4 g/L. Serum creatinine was 56.0 μmol/L, with eGFR of 125.6 mL/min/1.73 m^2^. Anti-PLA2R antibodies remain negative.

The absolute B-cell count had recovered to 28/μL, prompting an additional dose of rituximab (0.5 g). At the 1-year follow-up, renal function remained stable, with serum creatinine of 69 μmol/L. The 24-h urinary protein excretion had declined to 140 mg, and serum albumin had normalized to 44.4 g/L. An extended panel of 12 serum antibodies associated with membranous nephropathy (Table 2) was performed, all of which returned negative results. The patient continues to be under active follow-up.

**Table 2:** Detection results of membranous nephropathy antibody profile.

Item	Assay method	Result	Clinical significance and disease relevance (20)
Anti-phospholipase A2 receptor antibody (PLA2R)	CBA method	−	~80% of primary MN; primary diagnostic biomarker
Anti-thrombospondin type-1 domain-containing 7A antibody (THSD7A)	CBA method	−	~1–5% of primary MN; some cases associated with malignancy
Anti-neural cell adhesion molecule 1 antibody (NCAM1)	CBA method	−	~2% of primary MN; ~6.6% of positive cases have lupus nephritis
Anti-semaphorin 3B antibody (SEMA3B)	CBA method	−	~1% of primary MN; predominantly observed in children
Anti-neural epidermal growth factor-like 1 antibody (NELL-1)	CBA method	−	Associated with MN related to Indian herbal medicine or α-lipoic acid use
Anti-contactin 1 antibody (CNTN1)	CBA method	−	Associated with MN that may precede demyelinating polyneuropathy
Anti-proprotein convertase subtilisin/kexin type 6 antibody (PCSK6)	CBA method	−	Associated with MN in chronic NSAID users; often responds to drug withdrawal
Anti-FAT atypical cadherin 1 antibody (FAT1)	CBA method	−	Associated with MN following hematopoietic stem cell transplantation
Anti-HtrA serine peptidase 1 antibody (HTRA1)	CBA method	−	~4.2% of PLA2R/THSD7A/NELL-1/EXT2-negative primary MN
Anti-protocadherin 7 antibody (PCDH7)	CBA method	−	Associated with MN showing low or no complement activation
Anti-neurotrophic factor, neuronal (NDNF) antibody	CBA method	−	Found in MN patients with syphilis
Anti-netrin G1 antibody (NTNG1)	CBA method	−	<1% of MN cases; no specific systemic disease association

CBA, cell-based assay; MN, membranous nephropathy; NSAIDs, non-steroidal anti-inflammatory drugs.

## Discussion

Among renal glomerular injuries associated with AS, IgA nephropathy is the most common pathological type (3), whereas concomitant MN is exceedingly rare, with only 10 cases reported in PubMed to date (4, 5). MN, a common glomerular disease characterized by subepithelial immune complex deposition along the glomerular basement membrane, typically presents clinically as nephrotic syndrome or proteinuria (6). It is classified as primary or secondary, the latter often linked to infections, drugs, malignancies, or autoimmune diseases (7).

Whether MN represents a secondary manifestation of AS or a coincidental finding remains uncertain. Among the 10 previously reported cases of AS with MN, several confounding factors were present: 2 cases had concomitant rheumatoid arthritis and had received gold therapy; 1 had long-term etanercept use, a drug implicated in MN pathogenesis (8). Furthermore, nine patients had a history of long-term NSAID use, which has been suggested to potentially contribute to MN development (9). A Mendelian randomization analysis using large-scale genome-wide association data indicated no causal effect of genetically predicted AS on MN (10); however, this conclusion is limited by sample size and requires further validation.

In our case, the patient had no underlying comorbidities such as hypertension or diabetes other than long-standing AS. Although he had previously been treated with etanercept, NSAIDs, and sulfasalazine, these medications had been discontinued 9 years before the onset of MN. Sulfasalazine and etanercept were briefly reintroduced for 1 month and then discontinued again. Given the prolonged drug-free interval, pathological features are inconsistent with drug-induced MN (7), and negative serum PCSK6 unrelated to NSAID use (9), a drug-induced etiology appears unlikely but cannot be entirely excluded.

Furthermore, several laboratory findings argue against a primary MN diagnosis. Specifically, serological markers, such as anti-PLA2R and anti-THSD7A antibodies, were negative. Renal biopsy revealed electron-dense deposits in the mesangial areas, and immunohistochemistry for IgG4 was negative, collectively favoring a secondary MN etiology (7). After excluding common secondary causes of MN, including autoimmune diseases, infections, and malignancies, kidney injury related to AS remained a plausible consideration. Regrettably, PLA2R staining on renal tissue was not performed due to laboratory constraints, precluding further etiological clarification.

In the reported cases of AS-associated MN, all patients had a prolonged AS course. Only two documented cases reported AS disease activity using the BASDAI score, making it impossible to establish a statistical correlation between MN and AS activity. In our patient, who presented with typical MN symptoms such as fatigue and bilateral ankle edema, the BASDAI score was 2.60. CT scans showed only mild bilateral sacroiliitis (Figure 1), and he reported no typical AS symptoms such as morning stiffness or joint pain, suggesting low AS disease activity. In contrast, MN was highly active, evidenced by nephrotic-range proteinuria and hypoalbuminemia. Therefore, in agreement with Tohma et al. (4), we conclude that there is no apparent correlation between MN activity and AS disease activity.

Among previously reported cases, excluding two patients lost to follow-up and one death from myocardial infarction, three patients achieved renal remission after discontinuing relevant AS medications. The remaining four, after NSAID withdrawal, received various regimens, including pulse corticosteroids, corticosteroids combined with cyclophosphamide, or corticosteroids combined with cyclosporine, all with some therapeutic benefit.

In our case, the patient had been treated with etanercept (a TNF-*α* inhibitor) for 1 month before admission, without improvement in fatigue or edema. Considering the lack of correlation between MN and AS disease activity, further reduction of AS activity was deemed to have limited therapeutic value. Therefore, we did not initiate other anti-AS therapies such as adalimumab or new-generation therapies.

However, given the involvement of a major organ (the kidneys), along with nephrotic-range proteinuria (24-h urinary protein: 12.9 g), hypoalbuminemia, and hyperlipidemia, the patient faced increased risks of thrombosis and progression to end-stage renal disease (ESRD) without active intervention. After discussion with the patient and consideration of his preferences, a shared decision was made to initiate rituximab therapy in accordance with current clinical guidelines for membranous nephropathy.

Rituximab is a mouse–human chimeric IgG1κ monoclonal antibody targeting CD20 that depletes B cells through complement-dependent cytotoxicity (CDC), antibody-dependent cell-mediated cytotoxicity (ADCC), and direct induction of B-cell apoptosis (11–13). MN is considered an organ-specific autoimmune disease mediated by autoantibodies (6), and the pathogenesis of AS-associated MN may involve exposure of AS-related antigens in the subepithelial space of the glomerular basement membrane, forming *in situ* immune complexes with circulating antibodies (14). Therefore, rituximab, by depleting B cells and reducing autoantibody production, may play a therapeutic role in this condition.

Currently, rituximab is approved by the US FDA and European (FDA) and the European Medicines Agency (EMA) for non-Hodgkin lymphoma, rheumatoid arthritis, and ANCA-associated vasculitis and is used off-label in various autoimmune diseases (15). In recent years, it has become a first-line treatment option for primary MN. Multiple studies indicate that its efficacy is non-inferior or superior to traditional immunosuppressants, with a favorable safety profile. For instance, a study comparing RTX with cyclosporine A in idiopathic MN suggested that RTX rituximab had advantages in both efficacy and safety (16). Another retrospective cohort study showed that the rituximab treatment group had significantly fewer overall and serious adverse events compared with the steroid-plus-cyclophosphamide regimen (*p* < 0.001) (17). Furthermore, selective targeting of B cells offers a more specific immunomodulatory effect, potentially reducing infection risk.

For secondary MN, the dosage and regimen of rituximab require individualization. Common regimens include 375 mg/m^2^ weekly for 4 weeks, or 1 g administered in two divided doses (days 1 and 15) (18). Recent pharmacokinetic studies suggest that lower cumulative doses (e.g., 100 mg monthly for 6 months) might achieve similar B-cell depletion in some patients, potentially reducing cumulative risk (19). In this case, we used the classic “1 g on day 1 and day 15” regimen. At the 6-month follow-up, the patient showed normal renal function, 24-h urinary protein of 836 mg, and serum albumin of 35.4 g/L, meeting the KDIGO 2021 criteria for partial remission. As the peripheral B-cell count recovered to 28/μL, a supplemental dose of rituximab (0.5 g) was administered according to guideline recommendations. At the 1-year follow-up, renal function tests remained within the normal range. Follow-up for sustained efficacy is ongoing.

In conclusion, we report a rare case of AS combined with MN successfully treated with rituximab. One limitation is that PLA2R staining on renal tissue was not performed due to laboratory constraints, precluding further confirmation of the MN subtype. The causal relationship and pathogenesis between AS and MN remain unclear, and this case contributes additional clinical data in this area. For selected patients with AS-associated MN, particularly those with contraindications to corticosteroids or concerns regarding adverse effects of traditional immunosuppressants, rituximab may be considered a therapeutic alternative.

## Data Availability

The original contributions presented in the study are included in the article/supplementary material, further inquiries can be directed to the corresponding author.
